# Comigration Behavior of Cr(VI) and Microplastics and Remediation of Microplastics-Facilitated Cr(VI) Transportation in Saturated Porous Media

**DOI:** 10.3390/polym16233271

**Published:** 2024-11-24

**Authors:** Zijiang Yang, Yuheng Ma, Qi Jing, Zhongyu Ren

**Affiliations:** 1 Faculty of Architecture, Civil and Transportation Engineering, Beijing University of Technology, Beijing 100124, China; 2 Jianghe Water Resources & Hydropower Consulting Center Co., Ltd., Beijing 100120, China

**Keywords:** Cr(VI), microplastics, saturated porous media, cotransport, remediation

## Abstract

The study of the co-transport of Cr(VI) and microplastics (MPs) in porous media is important for predicting migration behavior and for achieving pollution removal in natural soils and groundwater. In this work, the effect of MPs on Cr(VI) migration in saturated porous media was investigated at different ionic strengths (ISs) and pHs. The results showed that pH 7 and low IS (5 mM), respectively, promoted the movement of Cr(VI), which was further promoted by the presence of MPs. The Derjaguin–Landau–Verwey–Overbeek (DLVO) results showed that the repulsive energy barrier between MPs and quartz sand decreased with increasing IS and decreasing pH, respectively, which promoted the retention of MPs in quartz sand and constrained the competition of Cr(VI) for adsorption sites on the surface of the quartz sand, thus facilitating the enhanced migration of Cr(VI), while Cr(VI) behaved conversely. Sodium alginate/nano zero-valent iron-reduced graphene oxide (SA/NZVI-rGO) gel beads could achieve the removal of MPs through a π-π interaction, hydrogen bonding, and electrostatic attraction, but the MPs removal would be reduced by 40% due to the competitive adsorption of Cr(VI). Notably, 97% Cr(VI) removal could still be achieved by the gel beads in the presence of MPs. Therefore, the gel beads can be used as a permeation reaction barrier to inhibit the MP-induced high migration of Cr(VI). The Cr(VI) breakthrough curves in reactive migration were well-fitted with the two-site chemical nonequilibrium model. Overall, the findings of this work contribute to the understanding of the migration behavior of Cr(VI) and MPs in saturated porous media and provide a theoretical basis for the remediation of soils and groundwater contaminated with Cr(VI) and MPs.

## 1. Introduction

Chromium (Cr) is a representative heavy metal that exhibits a variety of oxidation states, varying from −2 to +6. Cr is present in large quantities in industrial wastewater from electroplating, steel industry, textile printing, and dyeing and consequently accumulates in soil and water [1]. The most commonly occurring and stable forms of chromium present in soil environments are trivalent chromium (Cr(III)) and hexavalent chromium (Cr(VI)) [2]. It is important to note that the toxicity and mobility of Cr(III) and Cr(VI) vary significantly, leading to divergent implications for human health and the surrounding natural ecosystem. Consequently, understanding these differences is crucial for assessing the environmental risks associated with chromium contamination [3]. In terms of toxicity, Cr(VI) is more hazardous to physiological and metabolic processes compared to trivalent chromium (Cr (III)) [4], and prolonged exposure to elevated concentrations of Cr(VI) may cause lesions such as carcinogenesis and malformations in cellular tissues [5]. In terms of mobility, Cr(III) can be adsorbed by the substrate and converted to a solid phase, making it difficult to dissolve. However, Cr(VI) is more stable and present in the dissolved state (CrO_4_^2−^, HCrO_4_^−^, and Cr_2_O_7_^2−^) in natural water bodies and has a higher migration capacity [6]. It was reported that at a chromate production site in central China, the average value of Cr(VI) in soil samples was 181 mg/kg, which exceeded the maximum concentration limit (78 mg/kg) stipulated in China’s Risk Control Standard for Soil Environmental Quality of Soil Pollution on Development Land and at the chromium slag dump site. The concentration of hexavalent chromium (Cr(VI)) in groundwater has been reported to reach an alarming level of 273 mg/L, significantly exceeding the United States Environmental Protection Agency (US EPA) drinking water standard of 0.05 mg/L by a factor of 5460 [7,8]. This substantial overage underscores the critical need to predict the migration behavior of Cr(VI) in subsurface environments, which is essential for the effective remediation of soils and groundwater contaminated by this toxic species. Previous studies have demonstrated that the fate of Cr(VI) in contaminated soil systems is strongly influenced by various hydrochemical parameters, including ionic strength (IS) and pH, as well as the presence of colloidal materials [9].

Microplastics (MPs) represent a newly emerging pollutant of significant global concern, increasingly contributing to a serious environmental challenge worldwide. The continued production and overconsumption of plastics have resulted in widespread plastic pollution [10]. Even though most plastics are recyclable materials, about 79% of them are landfilled or released into the natural environment [11]. Improper disposal can result in large amounts of plastic waste being broken down into MPs (1 μm–5 mm) that mix in the environment in various shapes, sizes, and polymers [12,13]. MPs can be ingested and subsequently accumulate within the food chain [14,15]; the exposure of aquatic animals or mammals to micro-nano plastics induces oxidative stress that may lead to cellular damage and increase the potential to cause neurotoxicity [16]. Agricultural disturbances, root growth, and soil animal and microbial communities (earthworms, larvae, vertebrates) have been found to cause surface and deep soil disturbance, thereby promoting microplastic migration in the soil [17,18]. In addition, MPs may end up in groundwater along the soil profile under the action of anthropogenic activities, (micro)biological activities, and leaching [19,20]. Currently, MPs have been found to be widespread not only in agricultural, industrial, and urban soils, but even in the Tibetan Plateau [21] and polar regions [22]. Several investigations have found that the migration of MPs in the environment is influenced by ionic strength (IS) [23] and pH [24], among others. However, there has been a scarcity of research concentrating on the interactions between microplastics and heavy metals within porous media.

Colloidal particles within porous media have been shown to either facilitate or restrict the migration of heavy metals [25,26]. Similar to other colloidal particles, MPs can also adsorb heavy metals from the environment, leading to combined contamination as a result of their significant hydrophobic properties and large specific surface area [27]. In this process, MPs act as carriers of other environmental contaminants to other organisms, which may cause more severe combined toxic effects [28,29]. It has been shown that MPs may comigrate with other heavy metals by forming heterogeneous aggregates during groundwater transport or inhibit heavy metal transport due to competitive sorption [20]. People found that the addition of microplastics reduced the sorption capacity of soil for Cd^2+^ and promoted the migration rate of Cd^2+^, increasing Cd^2+^ accumulation in crops [30]. Researchers also found that when Tl(I) coexisted with MPs, more Tl(I) was deposited in porous media and Tl(I) partially hindered the migration of nanoparticles/microplastics (NPs/MPs) in porous media [31]. However, to date, no comprehensive studies have been undertaken to investigate the impact of microplastics, functioning as artificial colloids, on the migration dynamics of hexavalent chromium (Cr(VI)). This gap in research represents a critical area of inquiry, given the increasing prevalence of microplastics in environmental systems and their potential role in facilitating the transport of contaminants like Cr(VI).

In recent years, Cr(VI) removal techniques have been relatively well-established and comprehensive [32,33], Nonetheless, adsorption techniques for microplastics remain in the exploratory phase [34]. Owing to its considerable specific surface area and elevated reactivity [35], nano zero-valent iron (NZVI) has been extensively employed as an effective agent for the remediation of heavy metals in groundwater systems. This unique combination of characteristics enables NZVI to facilitate the reduction and immobilization of various heavy metal contaminants, making it a valuable tool in environmental cleanup efforts [36,37,38,39]. In addition, many studies have successfully applied graphene porous materials for the adsorption of MPs [40,41,42,43] and heavy metals [44]. However, it is important to note that NZVI tends to reduce its specific surface area due to aggregation into larger particles, driven by magnetic and van der Waals forces [45]. This aggregation can hinder its effectiveness as an adsorbent, underscoring the need for strategies to mitigate particle agglomeration in practical applications. Due to the high reactivity, NZVI will gradually oxidize and corrode [46]. However, the loading of NZVI on graphene can effectively solve the above problems [47,48]. In addition, nanopowder materials are difficult to collect and separate after being applied to water pollution remediation treatment, which is potentially leading to secondary pollution in the environment. Based on such problems, many studies have used natural non-toxic products such as alginate to cover nanomaterials to prepare hydrogel beads for reuse and recycling [49,50]. However, alginate gel beads are subject to swelling under specific conditions, leading to limited use of hydrogels [51], but the introduction of reduced graphene oxide to form a double lattice structure can enhance the mechanical characteristics of alginate beads [52]. At the same time, rGO and SA will form interfacial hydrogen bonds, so that the SA and rGO surfaces are tightly bonded [53]. Moreover, rGO and SA can generate π-electron clouds in the conjugated system, which further promotes the close bonding of rGO and SA [54]. Therefore, the gel beads formed by coating the composite with sodium alginate are expected to achieve the removal of Cr(VI) and MPs, and the gel beads are easily recyclable after application.

Therefore, the aims of this study were (I) to investigate the effect of MPs on the migration behavior of Cr(VI) in porous media considering the action of hydrochemical conditions and (II) also, to investigate the feasibility of applying sodium alginate/nano zero-valent iron-reduced graphene oxide (SA/NZVI-rGO) gel beads as an adsorbent to remove Cr(VI) and MPs from porous media. In our previous research work [55], the synthesized SA/NZVI-rGO gel beads exhibited sufficient stability, enhanced mechanical properties, and effective removal of Cr(VI). Based on these research objectives, we carried out the following research works, firstly, to explore the effect of MPs (i.e., 1 μm polystyrene microspheres) on Cr(VI) migration in quartz sand media at IS of 5 and 25 mM. Secondly, comigration experiments of Cr(VI) and MPs in quartz sand columns were conducted in 5 mM NaCl at pH 5 and 7. In addition, the effect of gel beads as a reaction barrier on the migration of Cr(VI) in saturated porous media under the coexistence system was carried out based on the performance of SA/NZVI-rGO gel beads on the removal of Cr(VI) and MPs in the separate and coexistence systems verified by batch experiments. Finally, to evaluate the reactive migration of Cr(VI) in saturated porous media under the coexistence system, a two-point nonequilibrium model was used to fit the measured data of Cr(VI). This work uncovers the migration behavior of Cr(VI) in saturated porous media under the potential threat of MPs. The findings of this study provide a theoretical framework that can inform remediation strategies for Cr(VI)-contaminated soils and groundwater, enhanced by the presence of microplastics as potential facilitators of contaminant transport.

## 2. Materials and Methods

### 2.1. Materials

Potassium dichromate purchased from Tianjin Fuchen Chemical Reagent Co., Ltd, Tianjin, China. was used to prepare the stock solution of Cr(VI). In all experiments, the target concentration of Cr(VI) (20 mg/L) was established through the dilution of the stock solution using deionized water. The effluent Cr(VI) content was measured with an ultraviolet spectrophotometer (2802S UV/VIS, Shanghai Unicosh Instruments Co. Shanghai, China), and the detailed measurement protocol is given in Text S1. As representative MPs, 1.0 μm green fluorescent polystyrene microspheres were used (Tianjin Baseline Chromtech Research Centre, Tianjin, China). In all experiments, the MP suspension was diluted to 5 mg/L with deionized water. The MP suspension was stored in brown glassware in the dark at 4 °C. The concentration of plastic particles was determined by a fluorescence spectrophotometer (F-7000, Hitachi High-Technologies, Japan). For more detailed information on MPs, Texts S2 and S3. The mean hydrodynamic diameter and zeta potential of the plastic particles required for the experiments were obtained by dynamic light scattering (DLS) and electrophoretic mobility using a zeta potential analyzer (90 Plus, Brookhaven, GA, USA). These results are shown in Table S1. All reagents and solvents employed in this study were of analytical grade. Quartz sand (No. S861671-500g, Shanghai Maclean Biochemical Co., Ltd., Shanghai, China) was used as the porous medium in this work with a size range of 16 to 30 mesh (0.55–1 mm). Sodium alginate/nano zero-valent iron-reduced graphene oxide (SA/NZVI-rGO) gel beads were synthesized and characterized in our previous publication [55].

### 2.2. Column and Batch Experiments

Porous media (quartz sand without and with SA/NZVI-rGO) was filled into cylindrical Plexiglas columns (inner diameter 2 cm × length 14 cm). The experimental setup is shown in Figure S4, and the details of the column filling method are given in Text S4 and the column parameters in Text S5 and Tables S2 and S3. After filling, the background electrolyte solution (5 PV, pore volume) with the desired pH and ionic strength was pumped into the column for pre-equilibration. After pre-equilibration, 8 PV suspensions (Cr(VI), MPs, or a mixture of Cr(VI) and MPs) were injected into the column. During the migration experiments, the MP suspension was sonicated periodically to maintain stability. Finally, the target solute was eluted with 5 PV of background electrolyte solution. During all migration experiments, a constant pore water flow rate was set to 1 mL/min (0.318 cm/min) and pumped in the upward flow direction by a BT-100-1F peristaltic pump (Baoding Lange Constant Flow Pump Co., Ltd. Baoding, China). Before the column experiment, the influent suspension was adjusted to a suitable pH with 1% HCl and 1% NaOH. The effluent samples were collected in brown glass vials and analyzed for concentration to obtain breakthrough curves. The quartz sand was also divided into 4 sections and used to generate retention profiles, and detailed overall recoveries (mass balance) for each experiment are shown in Text S6. In addition, batch experiments were used to determine the removal of Cr(VI) and MPs by SA/NZVI-rGO gel beads in single and composite systems, respectively (Texts S7 and S8), so as to investigate the removal of Cr(VI) by SA/NZVI-rGO gel beads, which served as a permeation barrier in quartz sand columns during composite contamination.

### 2.3. DLVO Interaction Energy Calculations

The classical Derjaguin–Landau–Verwey–Overbeek (DLVO) theory, which provides a comprehensive understanding of the stability of colloidal solutions, is widely employed to describe interactions between particles and surfaces [56]. This theory facilitates the prediction of the overall interaction energy, encompassing both van der Waals forces and electrostatic double-layer effects between microplastics and quartz sand. By applying the DLVO framework, researchers can gain deeper insights into the movement characteristics of plastic particles within saturated porous media, ultimately contributing to a better understanding of their transport and behavior in environmental contexts. The specifics of the DLVO calculation methodology are described in Texts S9 and S10.

### 2.4. Mathematical Modeling of Cr(VI) Reactive Transport

To validate the reactive transport of hexavalent chromium (Cr(VI)) within permeation barriers, breakthrough data were simulated using the HYDRUS-1D (Version 5.01) software. Convective diffusion equations are often employed to model metal ion transport; however, the reactive transport of solutes in porous media can be affected by a range of nonequilibrium phenomena [57]. These phenomena may include factors such as adsorption–desorption processes, chemical reactions, and hydrodynamic dispersion, which can significantly affect the migration patterns of solutes like Cr(VI) in subsurface environments. Therefore, in this work, the Cr(VI) breakthrough curves were simulated using a modified Richards equation [58] and a two-point adsorption model [59]. The modified Richards equation is described by (Equations (1) and (2)), as follows:

(1) $$\frac{\partial \theta }{\partial t}=\frac{\partial }{\partial x}\left[K\left(\frac{\partial h}{\partial x}+\cos\alpha \right)\right]-S$$

(2) $$K\left(h,x\right)=K_{S}\left(x\right)K_{r}\left(h,x\right)$$

The pressure head (cm) is represented as *h*, and *θ* denotes the volumetric water content. Time is indicated in minutes *t*, and *S* (min^−1^) refers to the source–sink function. The angle *α*, defined as the angle between the direction of flow and the vertical axis, is set at 0° for this research. The vertical coordinate *x* (cm) is oriented with the upward direction defined as positive. The unsaturated hydraulic conductivity is represented by *K* (cm/min), relative hydraulic conductivity is denoted as *Kr* (cm/min), and saturated hydraulic conductivity is indicated as *Ks* (cm/min). The flow of water was maintained at a stable rate, and the composite porous media was thoroughly saturated. The lower boundary of the system was designated as a constant flow boundary, ensuring consistent hydraulic conditions, while the upper boundary was treated as a specified head boundary, allowing for controlled water levels at the surface. This configuration is essential for accurately modeling the hydrological behavior within the porous media.

The solute transport model incorporates a two-site adsorption framework, which represents a chemical nonequilibrium scenario. In this model, the adsorption sites are categorized into two distinct types: the first type of site (S1) is considered instantaneous, while at the second type of site (S2), the adsorption is considered time-dependent. The equation of the two-site model is described as follows:

(3) $$\left(1+\frac{f\cdot \rho }{\theta }\left[\frac{b\cdot K_{L}}{{\left(1+K_{L}\cdot c\right)}^{2}}\right]\right)\cdot \frac{\partial c}{\partial t}=D\frac{\partial^{2}c}{\partial x^{2}}-v\frac{\partial c}{\partial x}-\frac{\alpha \cdot \rho }{\theta }\cdot \left[\left(1-f\right)\cdot \frac{b\cdot K_{L}\cdot c}{1+K_{L}\cdot c}-S_{2}\right]$$

In this context, *f* represents the equilibrium site fraction, while *α* (min^−1^) represents the first-order kinetic rate constant, *K_L_* (L/mg) denotes the Langmuir adsorption constant, and *b* (mg/g) signifies the maximum adsorption potential of the medium for the solute. S2 refers to the solid-phase concentration at type 2 sites, while *θ* indicates volumetric water capacity. The solute concentration in the aqueous phase is given by *c* (mg/L), *D* (cm^2^/min) represents the dispersion coefficient, *v* (cm/min) denotes pore water velocity, *x* (cm) is the spatial position, *ρ* (g/cm^3^) indicates the overall density of the porous media, and t signifies time. HYDRUS-1D necessitates input parameters including q (where q = Q/A), *θ*, λ (λ = *D·v*), *b*, *K_L_*, *f*, and *α* to construct a nonlinear non-equilibrium model (NLNE). The solute concentration at every position in the column is initially assumed to be zero. The lower boundary is defined as the concentration boundary, allowing for the introduction of solutes, while the upper boundary is treated as a flux boundary, facilitating the flow of solutes out of the system. This configuration establishes the foundational conditions necessary for modeling solute transport dynamics within the column.

## 3. Results and Discussion

### 3.1. Effect of IS on the Cotransport of Cr(VI) and Microplastics

**Figure 1:**
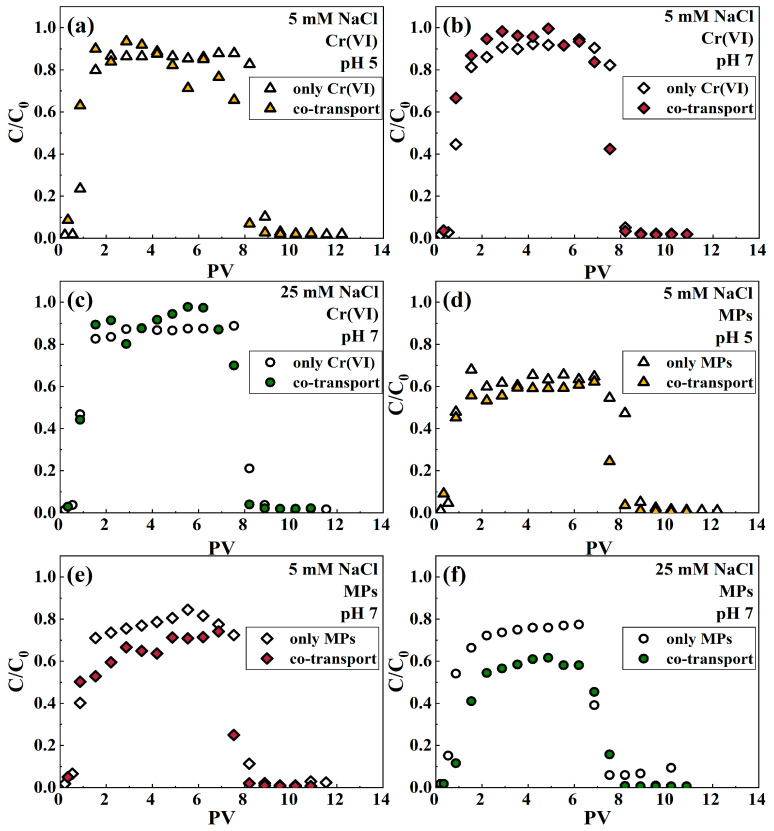
Breakthrough curves of Cr(VI) in both the absence and presence of MPs at pH 5 (**a**), pH 7 (**b**) with IS 5 mM, and pH 7 with IS 25 mM (**c**). Breakthrough curves of MPs in the presence and absence of Cr(VI) at pH 5 (**d**), pH 7 (**e**) with IS 5 mM, and pH 7 with IS 25 mM (**f**).

The effect of IS on the migration of MPs and Cr(VI) alone in a saturated quartz sand column was explored using NaCl as an electrolyte in suspension. In the context of Cr(VI) migration alone (Figure S11a), the breakthrough curve of Cr(VI) is slightly higher at low IS (5 mM) relative to high IS (25 mM), which implies that low ionic strength facilitates the movement of Cr(VI) in quartz sand. This is because the negative surface potential of quartz sand decreases with increasing ionic strength due to the neutralization reaction with Na^+^ (Table S1). As a consequence, the electrostatic repulsion between quartz sand and hexavalent chromium (Cr(VI)) diminishes, resulting in increased retention of Cr(VI) within the quartz sand at elevated ionic strength. This observation is consistent with previous findings, which highlight the influence of ionic strength on the adsorption behavior of contaminants in porous media [60]. Similarly, in the system of MP migration alone, Na^+^ can adsorb on the surface of negatively charged MPs and weaken their surface electronegativity by electrostatic interaction (Table S1), resulting in a weaker electrostatic repulsion between microplastics and quartz sand. Therefore, with the increase in IS, the breakthrough curve of MPs at IS = 25 mM is slightly lower than the breakthrough curve of MPs at IS = 5 mM (Figure S12a), which means more MPs are retained in the quartz sand, and the retention rates of MPs in quartz sand are 14.15% and 5.61% (Table S4), respectively. This is consistent with the results of the DLVO interaction energy calculation (Figure 1a), where the repulsion energy barrier of MP quartz sand decreases from 118.86 KT to −0.48 KT with the increase in IS. This trend suggests that the high-intensity background electrolyte inhibits the migration of MPs in the quartz sand column, aligning with findings from other research [61].

In comparison to the mono migration of hexavalent chromium (Cr(VI)), the breakthrough curves for Cr(VI) in the presence of microplastics (MPs) were significantly elevated under both ionic strength (IS) conditions (Figure 1b,c). These findings are clear from the reduction in retention capacity presented in Table S4. Specifically, under identical chemical conditions, the retention capacity of Cr(VI) within the binary system was measured at 2.02% and 1.59% for ionic strengths of 5 mM and 25 mM, respectively. In contrast, the retention capacity of Cr(VI) in the separate system was higher, at 2.09% and 2.15%, respectively. These results underscore the impact of MPs on the retention dynamics of Cr(VI) in porous media. These results suggest that relatively less Cr(VI) is adsorbed on the surface of quartz sand in the presence of microplastics, which enhances the mobility of Cr(VI). It was similarly found in the comigration system of Cr(VI) and bentonite colloidal particles, which enhanced the movement of Cr(VI) in saturated porous media [62]. In contrast, the presence of Cr(VI) in both IS solutions at pH 7 restricted the migration of MPs in quartz sand (Figure 1e,f). Moreover, the inhibition of the migration of MPs by Cr(VI) was enhanced with increasing IS (Figure S12c). As shown in Table S4, the recovery of microplastics (MPs) in the effluent passing through quartz sand decreased with increasing ionic strength, falling from 56.06% at 5 mM to 43.77% at 25 mM. This trend suggests that higher ionic strengths may enhance interactions between MPs and the quartz sand, thereby reducing the mobility and recovery of MPs in the effluent. On the other hand, as the IS increased, it was observed that the migration pattern of MPs in the comigrated system was similar to that in the single system. In all NaCl solutions at pH 7, when IS was the same, the breakthrough curves of Cr(VI) or MPs showed the same trend for both single and coexisting systems (Figures S11 and S12). These findings indicate that the interaction between Cr(VI) and MPs influences their migration behavior in saturated quartz sand to a certain degree.

**Figure 2:**
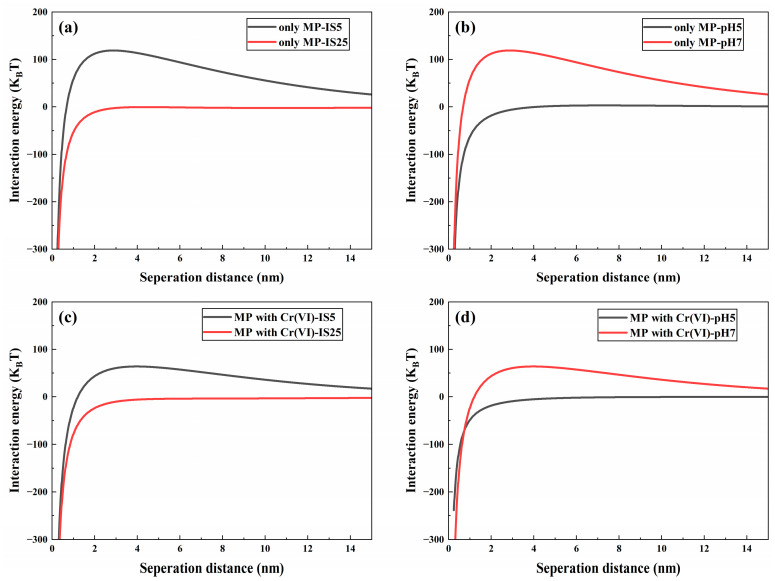
Interaction energy between MPs and quartz sand without Cr(VI) under different IS and same pH (**a**), MPs and quartz sand without Cr(VI) under same IS and different pH (**b**), MPs and quartz sand with Cr(VI) under different IS and same pH (**c**), MPs and quartz sand with Cr(VI) under same IS and different pH (**d**).

According to the recoveries of Cr(VI) and MPs (Table S4), it is clear that the retention of MPs by quartz sand is significantly greater than that of Cr(VI) under the same solution conditions. This disparity suggests that competition for adsorption sites on the surface of quartz sand between MPs and Cr(VI) could explain the elevated effluent concentrations of Cr(VI). Furthermore, the increased efflux of Cr(VI) in the presence of MPs may also result from the formation of Cr(VI)-MP aggregates [63], which could occupy available adsorption sites on the quartz sand, thereby impeding the retention of Cr(VI) within the quartz sand column [23,64]. However, the adsorption of Cr(VI) by polystyrene MPs was weak, with an average saturation adsorption capacity of 146.11 μg/g [65]. Consequently, the formation of Cr(VI)-microplastics (MPs) aggregates may not be a contributing factor to the increased migration of Cr(VI) influenced by MPs. From the perspective of MPs, the presence of Cr(VI) reduces the negative charge carried by the MPs (Table S1), which subsequently weakens the electrostatic repulsive forces between the MPs and the quartz surface. This reduction in repulsion facilitates greater retention of MPs within the system, aligning with the calculations derived from the Derjaguin–Landau–Verwey–Overbeek (DLVO) interaction energy model. Compared with the energy profile when MPs migrate alone, the energy barrier of MP deposition on the quartz sand surface decreases in the presence of Cr(VI) (Figure S10b,c). Additionally, the zeta potential of both microplastics and quartz sand decreased with increasing ionic strength IS (Table S1). This trend indicates that a higher concentration of Na^+^ ions gradually compresses the electric double layer surrounding the particles, while a high ionic strength creates more favorable conditions for the deposition of microplastics onto porous media. This is also consistent with the outcomes of DLVO interaction energy assessments (Figure 2c), where the MP-quartz sand repulsion energy barrier decreases with increasing IS in the coexistence system.

### 3.2. Effect of pH on the Cotransport of Cr(VI) and Microplastics

To explore the effect of pH on the migration of Cr(VI) and MPs in quartz sand media, the migration of single and dual systems at pH 5 and 7 were considered in a 5 mM NaCl background solution, respectively. In the monosystem, the overall breakthrough curve for hexavalent chromium (Cr(VI)) in acidic solution was observed to be slightly lower than that in a neutral solution (Figure S11b); this observation implies that acidic conditions may inhibit the migration of Cr(VI) through quartz sand. Such inhibitory effects could be attributed to changes in the chemical speciation of Cr(VI) and enhanced adsorption mechanisms under acidic pH conditions. These findings are comparable to those of previous studies [66]. The hydroxyl groups present on the surface of quartz sand can be protonated at lower pH (≡Si–OH + H+ → ≡Si–OH_2_^+^) [67], and Cr(VI) exists mainly as HCrO_4_^−^ and Cr_2_O_7_^2−^ at low pH [68]. Therefore, the adsorption of the positively charged ≡Si–OH_2_^+^ sites to Cr(VI) increases at pH 5. In other words, the decrease in negative charge on the quartz surface at pH 5 leads to a weaker electrostatic repulsion between quartz and Cr(VI), which inhibits Cr(VI) migration. Similarly, the effect of pH on the migration of MPs in individual systems is similar to that of Cr(VI). In the 5 mM NaCl background solution, the C/C_0_ of MPs decreased from approximately 0.84 under neutral conditions to 0.65 under acidic conditions (Figure S12b). This means that at pH 5, more MPs can be adsorbed on the media. This is because the lower zeta potentials of quartz sand and MP under acidic conditions lead to a weaker electrostatic repulsion between them (Table S1). This is consistent with the findings of the DLVO interaction energy analysis calculation (Figure 2b), where the repulsion energy barrier of MP-quartz sand decreases with decreasing pH. This trend implies that low pH promotes the retention of MPs in the quartz sand, which is consistent with the results of other studies [20].

As can be seen in Figure 1a,b, the presence of MPs promoted the migration of Cr(VI) under both pH levels compared to the migration system alone. These observations are clear based on the increased Cr(VI) outflow capacity shown in Table S4. The outflow capacity of Cr(VI) in the binary system (82.74% and 87.52% for pH 5 and 7, respectively) was higher than that of Cr(VI) in the mono system (81.88% and 86.11%, respectively) at the same IS. This phenomenon may be attributed to the competition between microplastics (MPs) and hexavalent chromium (Cr(VI)) for adsorption sites on the surface of quartz sand, leading to increased efflux of Cr(VI). On the contrary, the presence of Cr(VI) inhibited the movement of MPs in quartz sand in both pH solutions of IS 5 (Figure 1d,e). When MPs and Cr(VI) coexist, the negative charge on the surface of microplastics is reduced (Table S1), which diminishes the electrostatic repulsive force between MPs and quartz sand. This reduction facilitates greater retention of MPs within the porous media. Compared with the energy profile when MPs migrate alone, the energy barrier of MP deposition on the quartz sand surface decreases in the presence of Cr(VI) (Figure S10a,b). All of these prove that the presence of Cr(VI) promotes the retention of MPs in quartz sand. Meanwhile, it is more intuitive from Figures S11d and S12d that the acidic environments are more conducive to the retention of Cr(VI) and MPs in the binary system, which is the same trend as the migration of Cr(VI) and MPs in the mono system.

### 3.3. Effects of SA/NZVI-rGO Gel Beads on Cr(VI) Transport with Microplastics

The presence of MPs promotes the migration of Cr(VI) in quartz sand columns, leading to Cr(VI) entering deeper soil or even groundwater, so it is necessary to provide a permeable barrier to quartz sand to form a composite porous medium to inhibit MP-induced high migration of Cr(VI).

#### 3.3.1. The Removal of Cr(VI) and MPs by Gel Beads and an Analysis of the Mechanism

In a previous study, we successfully synthesized highly stable and mechanically enhanced sodium alginate/nano zero-valent iron/reduced graphene oxide (SA/NZVI-rGO) gel beads that effectively remove hexavalent chromium (Cr(VI)) from aqueous solutions [55]. To explore the potential of SA/NZVI-rGO gel beads as a permeation barrier when Cr(VI) and microplastics (MPs) co-migrate, we first evaluated their removal efficacy in a co-contaminated system of Cr(VI) and MPs through batch experiments. In the case of the separate system for Cr(VI), the SA/NZVI-rGO gel beads demonstrated a greater affinity for removing Cr(VI) under acidic conditions compared to neutral solutions (Figure S7). Specifically, the removal efficiency of Cr(VI) at pH 5 was consistently higher than that of Cr(VI) at pH 7, for the detailed reasons we have discussed in our previous article [55]. The difference in removal efficiency at adsorption equilibrium was about 1% at different IS (Figure S7), which indicates that IS is not the main factor affecting the removal of Cr(VI) by SA/NZVI-rGO gel beads. When Cr(VI) and MPs were co-contaminated (pH 5 IS 5 mM), although the removal efficiency of Cr(VI) of the single system was higher than that of Cr(VI) of the combined system in the former 120 min, the removal efficiency of Cr(VI) in the combined system (97.92%) surpassed that of the single system (96.40%). This finding suggests that the presence of MPs may enhance the overall adsorption process over time, leading to improved removal of Cr(VI) when both contaminants are present (Figure S7). This indicates that the presence of MPs did not affect the efficient removal of Cr(VI) by SA/NZVI-rGO gel beads.

In the case of MP contamination only (Figure S8), the low pH was more favorable for the removal of MPs by SA/NZVI-rGO gel beads relative to the neutral background solution, and the removal rate was increased from 71.10% to 81.55%. The observed decrease in electrostatic repulsion forces between sodium alginate/nano zero-valent iron/reduced graphene oxide (SA/NZVI-rGO) gel beads and microplastics (MPs) can be attributed to the neutralization of the negative charge on the gel beads by H^+^ ions. This reduction in negative charge facilitated increased adsorption of MPs onto the SA/NZVI-rGO gel beads, ultimately enhancing the removal efficiency of MPs in the co-contaminated system. At different ISs, the difference in the removal efficiency of MPs by SA/NZVI-rGO gel beads at adsorption equilibrium was not significant (70.62% and 70.84% at IS 5 mM and 25 mM, respectively), indicating that IS was not the main factor affecting the removal of MPs by SA/NZVI-rGO gel beads. On the contrary, compared to the removal efficiency of MPs in the single system (81.55%), the removal efficiency of MPs could only reach 38.92% in the Cr(VI) and MP co-contaminated systems. Conclusively, although the removal of MPs by SA/NZVI-rGO gel beads was not ideal during co-contamination, the presence of MPs did not affect the efficient removal of Cr(VI) by SA/NZVI-rGO gel beads, which could serve as a permeation barrier to inhibit the high Cr(VI) migration promoted by MPs.

**Figure 3:**
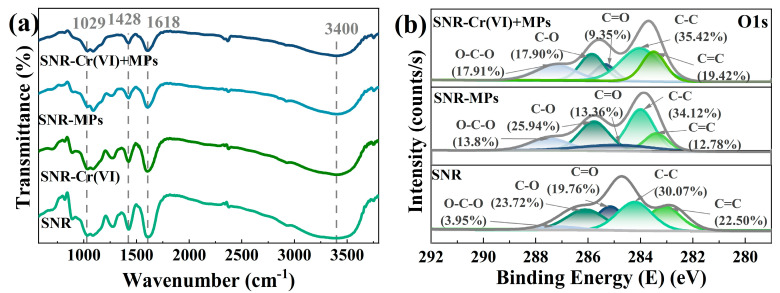
FTIR (**a**) and XPS-C1s spectra (**b**) of SA/NZVI-rGO (SNR) gel beads for Cr(VI) and MP removal and co-removal, respectively.

The removal mechanism of SA/NZVI-rGO gel beads for Cr(VI) and MPs was explored. In the FTIR, as shown in Figure 3a, the broad band around 3400 cm^−1^ indicates the stretching vibration of O–H, and the characteristic absorption peaks appeared at 1618, 1428, and 1029 cm^−1^, which were attributed to the deformation oscillation peaks of C=O, O=C–O, and O–H, respectively. A comparison of SA/NZVI-rGO gel beads before and after the adsorption of MPs revealed that the broad peak of O–H located near 3400 cm^−1^ was narrowed and the intensity of the deformation vibration peak of O–H at 1029 cm^−1^ was weakened; meanwhile, the intensity of the deformation vibration peaks of the carboxyl group at 1428 cm^−1^ (O=C–O) and 1618 cm^−1^ (C=O) was lowered. This indicates the formation of hydrogen bonding interactions between SA/NZVI-rGO gel beads and MPs. From the molecular structure point of view, the hydrogen atoms of polystyrene can form hydrogen bonding interactions with the hydroxyl and carboxyl groups of SA/NZVI-rGO gel beads. In the XPS shown in Figure 3b, by comparing the C1s spectra before and after the removal of MPs by SA/NZVI-rGO gel beads, it was found that the molar ratio of C=C decreased from 22.50% to 12.78%. This indicates that the π-π interaction between π-electron-rich rGO and benzene ring-containing polystyrene MPs contributes to MP removal. Previous studies confirmed that the π-π interactions between GO or O–C_3_N_4_ and aromatic rings favor their adsorption of MPs [43]. From the zeta potentials of MPs (Table S1), it is evident that a slight electrostatic attraction exists between the positively charged sodium alginate/nano zero-valent iron/reduced graphene oxide (SA/NZVI-rGO) gel beads and the weakly negatively charged microplastics (MPs) under acidic conditions. This attraction may further promote the adsorption of MPs onto the gel beads, contributing to the enhanced removal efficiency observed in the co-contaminated system. Overall, π-π interactions, hydrogen bonding, and electrostatic attraction contributed to the adsorption of MPs by SA/NZVI-rGO gel beads.

The FTIR in Figure 3a demonstrated that the intensity of the peaks of each functional group of the SA/NZVI-rGO gel beads after the removal of Cr(VI) and MPs, respectively, was weakened compared with that of the SA/NZVI-rGO gel beads, and the intensity of the peaks was further weakened after the co-removal. This indicates that the SA/NZVI-rGO gel beads successfully facilitated the co-removal of hexavalent chromium (Cr(VI)) and microplastics (MPs). However, the removal efficiency of MPs in the co-removal system was significantly reduced compared to that in the single-removal system. By analyzing the C1s spectra (Figure 3b), a comparison between the SA/NZVI-rGO gel beads used for MPs removal and those employed for the co-removal of Cr(VI) and MPs revealed that the molar ratio of C=C increased from 12.78% to 19.42%. This increase suggests changes in the surface chemistry of the gel beads influenced by the co-presence of Cr(VI). This confirms the weakened contribution of π-π interactions to the adsorption of MPs upon co-removal. The possible reason for this is that Cr(VI), which was preferentially adsorbed on gel beads, was reduced to Cr(III) by the powerfully reducing NZVI to form Fe-Cr co-precipitates as well as Cr(OH)_3_, and these deposits, together with Cr(VI), occupied some of the surface adsorption sites and hindered the contact between the MPs and the SA/NZVI-rGO gel beads, which inhibited the adsorption of MPs. The reaction process is described by the reactions (Equations (4)–(6)):

(4) $$3{Fe}^{0}+{{Cr}_{2}O_{7}}^{2-}+14H^{+}\to 2{Cr}^{3+}+3{Fe}^{2+}+7H_{2}O$$

(5) $$6{Fe}^{2+}+{{Cr}_{2}O_{7}}^{2-}+14H^{+}\to 6{Fe}^{3+}+2{Cr}^{3+}+7H_{2}O$$

(6) $${Cr}^{3+}+3{OH}^{-}\to Cr(OH{)}_{3}↓$$

#### 3.3.2. Cr(VI) Migrates with Microplastics in the Quartz Sand Filled with Gel Beads

**Figure 4:**
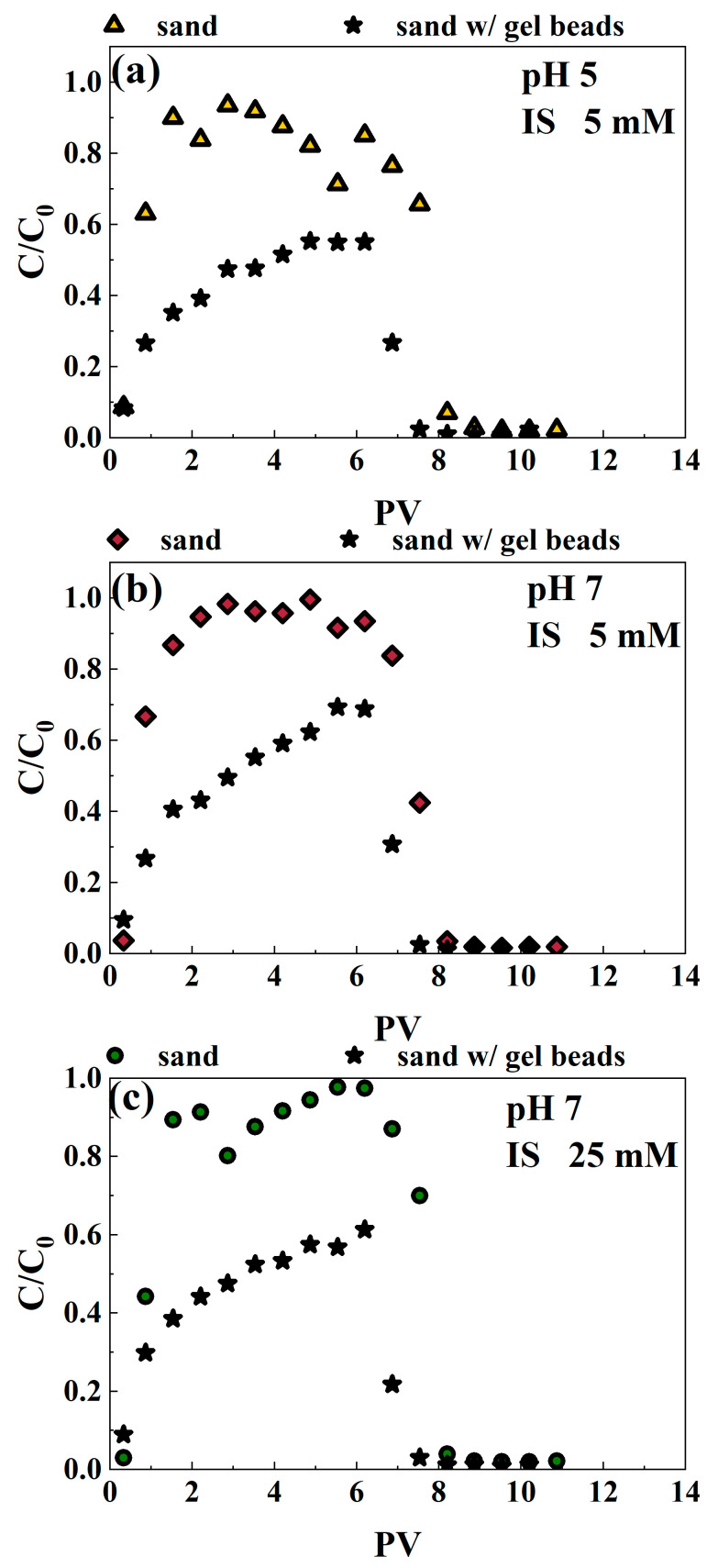
Breakthrough curves of Cr(VI) in quartz sand columns without and with SA/NZVI-rGO gel beads under comigration with MPs for three experimental conditions, pH = 5, IS = 5 mM (**a**), pH = 7, IS = 5 mM (**b**), pH = 7, IS = 25 mM (**c**). Here, “w/” refers to “with “.

To investigate whether the incorporation of SA/NZVI-rGO gel beads into quartz sand influences the migration of hexavalent chromium (Cr(VI)) in the presence of microplastics (MPs), migration experiments were conducted in NaCl solutions at pH levels of 5 and 7, under both low ionic strength (5 mM) and high ionic strength (25 mM) conditions. The breakthrough curves of Cr(VI) in pure quartz sand, without the addition of gel beads, were significantly elevated across all experimental conditions (Figure 4). This suggests that the presence of quartz sand alone facilitates the migration of Cr(VI), highlighting the need to assess the impact of gel beads on this transport process. Specifically, more than 82% of Cr(VI) (for all experimental conditions) passed through the quartz sand column (Table S4). However, the breakthrough curves of Cr(VI) in quartz sand columns containing gel beads were lower than those in pure quartz sand columns under all three experimental conditions (Figure 4). Specifically, the breakthrough ratios of Cr(VI) in pure quartz sand were 0.94, 0.99, and 0.98 for pH 5 IS 5 mM, pH 7 IS 5 mM, and pH 7 IS 25 mM solutions, respectively. In contrast, the corresponding breakthrough ratios decreased to 0.55, 0.69, and 0.61 with the incorporation of SA/NZVI-rGO gel beads into quartz sand. These observations indicate that the addition of SA/NZVI-rGO gel beads effectively reduces the movement of hexavalent chromium (Cr(VI)) in the presence of microplastics (MPs). This reduction in migration suggests that the gel beads may enhance retention mechanisms for Cr(VI), potentially improving the overall efficacy of the remediation process in contaminated environments.

### 3.4. Reactive Transport Model of Cr(VI) in Filled Saturated Porous Media During Comigration with MPs

**Figure 5:**
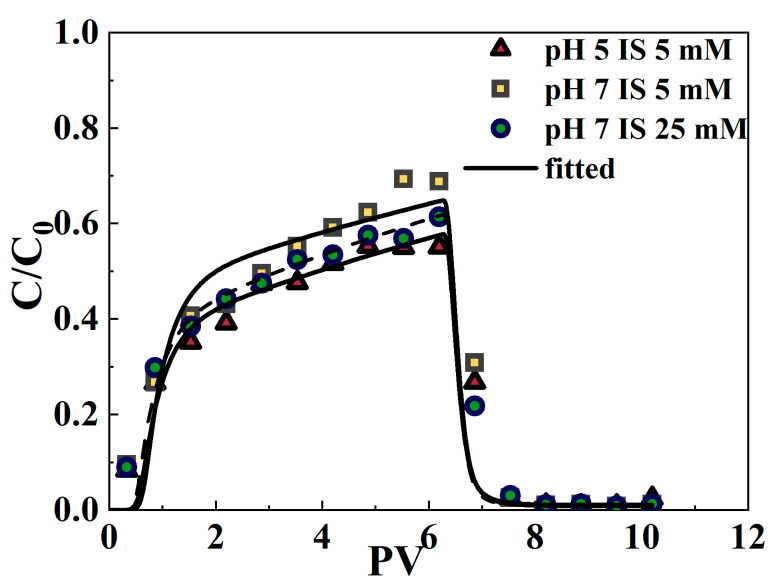
Breakthrough curves of Cr(VI) in quartz sand containing SA/NZVI-rGO gel beads under comigration with MPs for three experimental conditions. The symbols indicate experimental data, while the lines represent the fitted curve.

The isothermal experiments examining the adsorption of varying initial concentrations of Cr(VI) in a coexistence system using SA/NZVI-rGO gel beads and quartz sand are detailed in Text S8. From the fitted curves of isothermal adsorption (Figure S9) and the correlation coefficient R2 of isothermal adsorption (Table 1), considering the fitting results of different mediums comprehensively, it is evident that the Langmuir isothermal adsorption model provides a superior fit for the experimental data regarding the adsorption of Cr(VI) at various initial concentrations. In the case where the adsorption is described by the Langmuir equation, the two-site chemical nonequilibrium model was chosen to describe the reactive migration of the solute. For the equilibrium point, the Langmuir parameters (*b* and *KL*) from the isothermal adsorption experiments were used (Table 1), and the kinetic parameters (f and *a*) were determined by fitting the breakthrough data using the inverse solver module of HYDRUS-1D (Version 5.01). Figure 5 shows that the fitted breakthrough curves under the three experimental conditions can better simulate the trends of the measured data points. The root mean square error (RMSE) varies between 0.0554 and 0.0749, while the R^2^ values exceed 0.9303 (Table 2). This suggests that the two-site chemical nonequilibrium model in HYDRUS-1D effectively simulates the reactive migration of Cr(VI) in composite porous media within a coexistence system. Previous research has also shown that this model accurately fits the reactive transport of contaminants in porous media, enhancing the understanding of Cr(VI) transport mechanisms [57,69].

## 4. Conclusions

The results of this study showed that the electrostatic repulsion between quartz sand and Cr(VI)/MPs was weakened at high IS and low pH, respectively, resulting in more retention of Cr(VI) and MPs in saturated quartz sand. The DLVO results showed that when Cr(VI) was present, MPs were deposited more in quartz sand due to the reduction in the MP-quartz sand repulsion energy barrier, resulting in increased mobility of Cr(VI), which has a competitive adsorption relationship with MPs. SA/NZVI-rGO gel beads could remove Cr(VI) by electrostatic attraction, redox, and ion exchange as well as adsorb MPs by a π-π interaction, hydrogen bonding, and electrostatic attraction. For co-removal, SA/NZVI-rGO achieved 97% removal of Cr(VI), but MP removal was reduced from 81.55% to 38.92% due to competitive adsorption. The gel beads as permeation barriers effectively inhibited MP-facilitated high migration of Cr(VI), and their breakthrough curves could be well fitted by the two-site chemical nonequilibrium model (R^2^ > 0.93). Although the effects of pH and IS factors on Cr(VI) and MPs contamination were investigated in detail in this study, site experiments are needed to realize practical applications in the face of the complexity of actual soil and groundwater contamination situations. This work makes a significant contribution to understanding the synergistic migration of Cr(VI) and MPs in saturated porous media. Moreover, it provides a valuable theoretical foundation for the remediation of soil and groundwater contaminated with Cr(VI) and MPs.

## Figures and Tables

**Table 1:** Langmuir and Freundlich isotherm parameters for Cr(VI) removal by quartz sand and SA/NZVI-rGO gel beads under the coexistence system, respectively.

Medium	Condition	Langmuir Model			Freundlich Model		
		*K_L_* (L/mg)	*b* (mg/g)	R^2^	*K_F_* (L/g)	*n*	R^2^
Quartz Sand	pH 5 IS5	0.0023	0.3976	0.9406	0.0008	0.9844	0.9028
	pH 7 IS5	0.0025	0.3779	0.9425	0.0009	1.0148	0.8974
	pH 7 IS25	0.0026	0.3223	0.9610	0.0007	0.9819	0.8810
SA/NZVI-rGO gel beads	pH 5 IS5	0.1774	12.1309	0.9909	2.6411	2.2743	0.9683
	pH 7 IS5	0.1983	12.5629	0.9895	2.8576	2.2608	0.9944
	pH 7 IS25	0.2434	11.7162	0.9603	2.9654	2.3725	0.9667

**Table 2:** Inversion parameters of the two-site chemical nonequilibrium model.

Condition	Medium	*f*	*a* (min^−1^)	RMSE	R^2^
pH 5 IS5	Quartz Sand	0.0230 × 10^−1^	0.4283 × 10^−2^	0.0707	0.9425
	SA/NZVI-rGO gel beads	0.5750 × 10^−8^	0.1194 × 10^−5^		
pH 7 IS5	Quartz Sand	0.2973 × 10^−1^	0.4125 × 10^−2^	0.0749	0.9303
	SA/NZVI-rGO gel beads	0.1788 × 10^−4^	0.1591 × 10^−3^		
pH 7 IS25	Quartz Sand	0.2173 × 10^−1^	0.5358 × 10^−2^	0.0554	0.9671
	SA/NZVI-rGO gel beads	0.9645 × 10^−4^	0.1046 × 10^−3^		

## Data Availability

The original contributions presented in the study are included in the article/supplementary material; further inquiries can be directed to the corresponding author.

## Supplementary Materials

The following supporting information can be downloaded at https://www.mdpi.com/article/10.3390/polym16233271/s1: Text S1: The measurement of Cr(VI) concentration; Figure S1: UV spectrophotometer standard curve of Cr(VI) at a wavelength of 540 nm; Text S2: Detailed information of microplastics; Text S3: The measurement of nano(micro) plastics concentrations; Figure S2: Calibration curve of 1.0 μm MPs with fluorescence spectrophotometer at the excitation/emission wavelength of 488/518 nm; Figure S3: Fluorescence emission spectra of plastic particle and Cr(VI) at experimental concentrations; Table S1: Zeta potentials and hydrodynamic diameters of MPs, quartz, and SA/NZVI-rGO gel beads in experimental conditions; Text S4: Column Experiment; Figure S4: Schematic design of column device; Text S5: Dispersion Experiment; Figure S5: Standard curve for KCl; Figure S6: Breakthrough-leaching curves of KCl in quartz sand (a) and SA/NZVI-rGO (b); Table S2: Parameters of the packed quartz sand columns; Table S3: Parameters of the packed SA/NZVI-rGO gel beads columns; Text S6: Mass Recovery Protocol; Table S4: The mass balances of MPs and Cr(VI) in quartz sand column at experimental conditions; Text S7: Batch experiments; Figure S7: Removal efficiency of Cr(VI) in the absence and presence of MPs under different experimental conditions; Figure S8: Removal efficiency of MPs in the absence and presence of Cr(VI) under different experimental conditions; Text S8: Adsorption isotherm experiments; Figure S9: Langmuir and Freundlich isotherms for removing Cr(VI) under the coexistence system by SA/NZVI-rGO gel beads and quartz sand, respectively. Experimental conditions: concentration of Cr(VI) = 10–50 mg/L, concentration of MPs = 5 mg/L, and temperature = 16°C; Text S9: The calculation of the DLVO interaction; Text S10: Calculation of Hamaker constants; Figure S10: Interaction energy between MPs and quartz sand in the absence and presence of Cr(VI) at pH 5 IS 5 (a), pH 7 IS 5 (b), pH 7 IS 25 (c); Figure S11: Breakthrough curves of Cr(VI) at different IS(a) and different pH(b) in the absence of MPs and at different IS(c) and different pH(d) in the presence of MPs; Figure S12: Breakthrough curves of MPs at different IS(a) and different pH(b) in the absence of Cr(VI) and at different IS(c) and different pH(d) in the presence of Cr(VI). References [56,70,71,72] are included in the Supplementary Materials.
