# Supplementary material for: Comigration Behavior of Cr(VI) and Microplastics and Remediation of Microplastics-Facilitated Cr(VI) Transportation in Saturated Porous Media
Source: Polymers (Basel). 2024 Nov 24;16(23):3271. doi: 10.3390/polym16233271 (PMC11644253; doi:10.3390/polym16233271)
Supplement: Supplementary file 1 [file polymers-16-03271-s001.zip › polymers-3290653-supplementary.pdf]

Supplementary Information

Comigration Behavior of Cr(VI) and Microplastics and Remediation of Microplastics-Facilitated Cr(VI) Transportation in Saturated Porous Media

Zijiang Yang [1,†], Yuheng Ma [1,2,†], Qi Jing [1,*] and Zhongyu Ren [1]

[1] Faculty of Architecture, Civil and Transportation Engineering, Beijing University of Technology, Beijing 100124, China
[2] Jianghe Water Resources & Hydropower Consulting Center Co. Ltd., Beijing 100120, China
* Correspondence: jingqi@bjut.edu.cn; Tel.: +86-185-0195-00178
[†] These authors contributed equally to this work.

Text S1. The measurement of Cr(VI) concentration

For the effluent samples, the concentration of Cr(VI) was first measured after passing through a 0.22μm filter membrane, and the concentration of Cr(VI) was measured using the GB7467-87 diphenylcarbodihydrazide spectrophotometric method. The standard curve of Cr(VI) mass to absorbance was measured and plotted according to the requirements of this standard (Figure S1)。

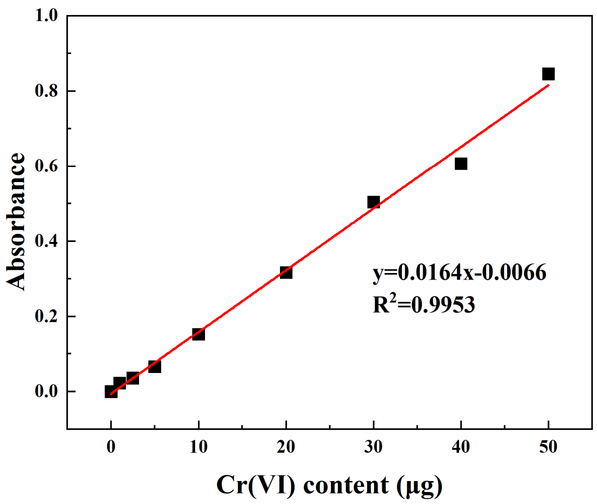

Figure S1. UV spectrophotometer standard curve of Cr(VI) at a wavelength of 540 nm.

Then 0.5mL H_2SO_4 solution (1+1) and 0.5mL H_3PO_4 solution (1+1) were added dropwise, followed by 2mL of color developer (diphenylcarbonyl dihydrazide and acetone solution), shaken well and left for 10min, and the absorbance was measured by UV spectrophotometer, and the concentration of Cr(VI) in the water sample was calculated by referring to the standard curve.

Text S2. Detailed information of microplastics

According to the manufacturer, the green fluorescent polystyrene microspheres (Tianjin Baseline Chromtech Research Centre) were prepared by combining fluorescent molecules on the surface of polystyrene. The fluorescent microspheres are characterized by high fluorescence intensity, stable performance and narrow particle size distribution, with a particle size of 1.0 μm and a concentration of 10 mg/mL. To

prepare the experimental suspension, the original suspension was first diluted with deionized water to 10 mg/L as a stock solution. The mixture was sonicated for 15 min, and then diluted with deionized water to a target concentration of 5 mg/L, while adjusting the pH and ionic strength.

Text S3. The measurement of nano(micro) plastics concentrations

All polystyrene microspheres have green fluorescence, so a fluorescence spectrophotometer (F-7000, Hitachi High Tech Science, Japan) and a 10 mm × 10 mm quartz cuvette were selected to analyze the concentration of MPs. The optimal excitation/emission wavelength was determined to be 488/518 nm, and both excitation and emission slits of the instrument were set to 5 nm. seven different concentrations of 1.0 μm MPs suspensions (0.1, 0.2, 0.5, 1, 2, 5 and 10 mg/L) were selected and prepared by diluting 10 mg/L MPs stock solution with deionized water to obtain the calibration curve (Figure S2). Based on the pre-established calibration curve for MPs, the effluent concentration of plastic particles was estimated.

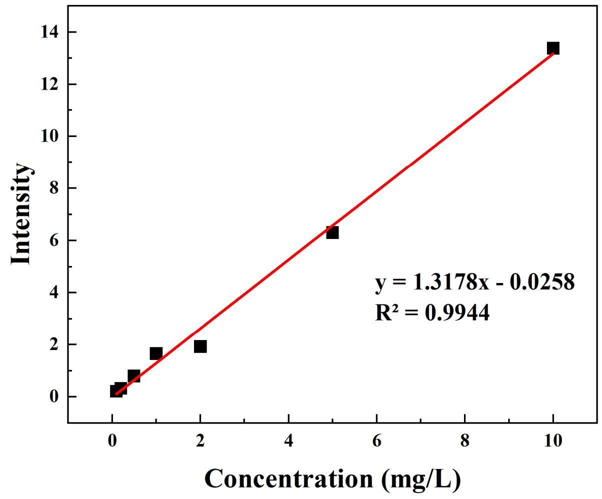

Figure S2. Calibration curve of 1.0μm MPs with fluorescence spectrophotometer at the excitation/emission wavelength of 488/518 nm.

More importantly, the above method is still applicable to measure the concentration of plastic particles even in the presence of Cr(VI) at the same time. To demonstrate this, the fluorescence intensity of the Cr(VI) solution (10 mg/L) was

measured at the corresponding wavelengths (λ em = 500 - 700 nm, λ ex = 488 nm). As shown in Figure S3, the fluorescence intensity values of Cr(VI) and background solutions were almost negligible. Therefore, the fluorescence spectrophotometer was able to accurately determine the concentration of MPs in this study even in the presence of Cr(VI) in the suspension.

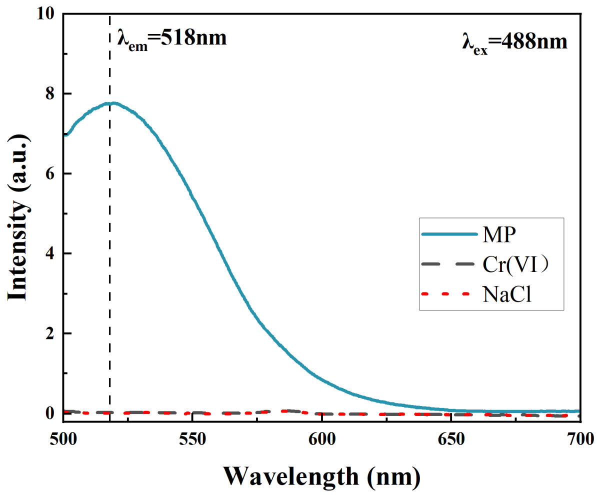

Figure S3. Fluorescence emission spectra of plastic particle and Cr(VI) at experimental concentrations.

Table S1. Zeta potentials and hydrodynamic diameters of MPs, quartz and SA/NZVI-rGO gel beads in experimental conditions.

Systems	pH	Ionic Strength (mM)	Zeta Potential (mV)	Hydrodynamic Diameter (nm)
MPs	5	5	-5.94±1.03	1132.76±17.85
	7	5	-11.22±1.69	1309.28±19.70
	7	25	-6.20±0.84	1229.61±33.67
MPs + Cr(VI)	5	5	-3.12±1.01	740.49±15.18
	7	5	-8.45±2.67	1196.06±26.58
	7	25	-4.61±3.18	1438.42±106.90
Quartz sand	5	5	-5.15±1.77	NA
	7	5	-22.68±1.41	
	7	25	-9.99±2.58	

NA represents for not analyzed.

The zeta potential of quartz sand was referenced in [70].

Text S4. Column Experiment

The migration-deposition behavior of Cr(VI) and MPs in porous media was simulated by a pure quartz sand column. A Plexiglas column (2 cm inner diameter × 14 cm length) was selected, and glass beads with a particle size of 3 mm were filled with 2 cm at the inlet and outlet respectively for ensuring the uniformity of the inlet water, and quartz sand was selected to constitute the aqueous medium of the sand column, with a quartz sand filling height of 8 cm.

The migration-transformation behavior of Cr(VI) was simulated by column experiment using SA/NZVI-rGO gel beads as permeation barrier. Glass beads with a particle size of 3 mm were filled with 2 cm at the inlet and outlet to ensure the uniformity of the inlet water, 1 cm of quartz sand was filled at the top and bottom, and SA/NZVI-rGO gel beads (6 cm) were filled in the sand layer as a permeation barrier in the middle layer.

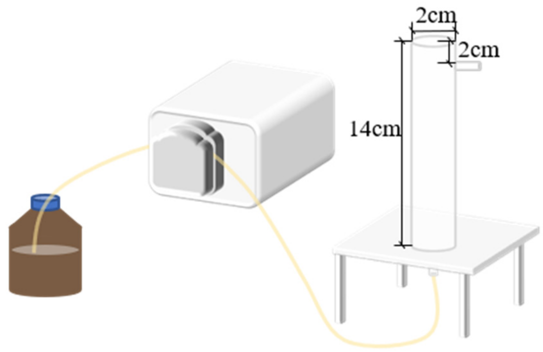

Figure S4. Schematic design of column device.

Text S5. Dispersion Experiment

The analytical solution method was used to calculate the dispersion coefficient, and KCl with good electrical conductivity was selected as the tracer to obtain the penetration curve. The conductivity method was used to determine the KCl concentration in the dispersion test. Different concentrations of KCl solutions were prepared separately, and the conductivity values were measured by a HACH portable

conductivity meter, and the conductivity-concentration relationship curves were shown in Figure S5, with excellent linear correlation between both.

The sand column was filled with quartz sand (or SA/NZVI-rGO) of height X. The 0.01 mol/L KCl solution was continuously injected into the saturated sand column, and the conductivity values were measured by taking samples in the sampling holes at regular intervals, and after the conductivity values remained stable, the sand column was rinsed with deionized water until the KCl concentration was stable to end the test. The KCl penetration rinse curve was plotted (Figure S6). The dispersion coefficient was calculated as follows:

$$D=\frac{1}{8}\left[\frac{X-U\bullet t_{0.16}}{\sqrt{t_{0.16}}}-\frac{X-U\bullet t_{0.84}}{\sqrt{t_{0.84}}}\right] \tag{S1}$$

where D (cm^2/min) is the dispersion coefficient, X (cm) is the coordinate of the calculation point, U (cm/min) is the velocity of pore water in the column, $t_{0.16}$ is the time corresponding to a C/C0 of 0.16 and $t_{0.84}$ is the time corresponding to a C/C0 of 0.84.

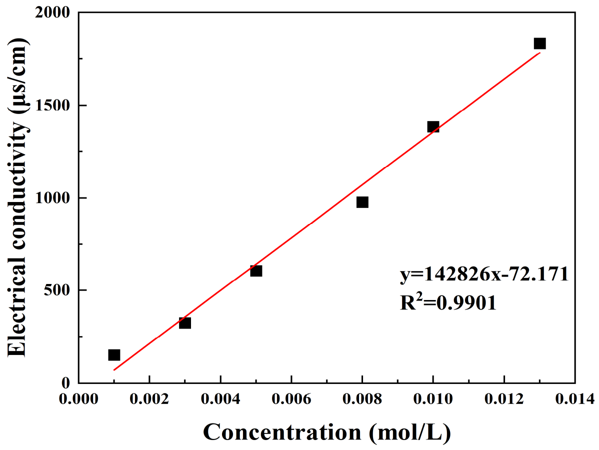

Figure S5. Standard curve for KCl.

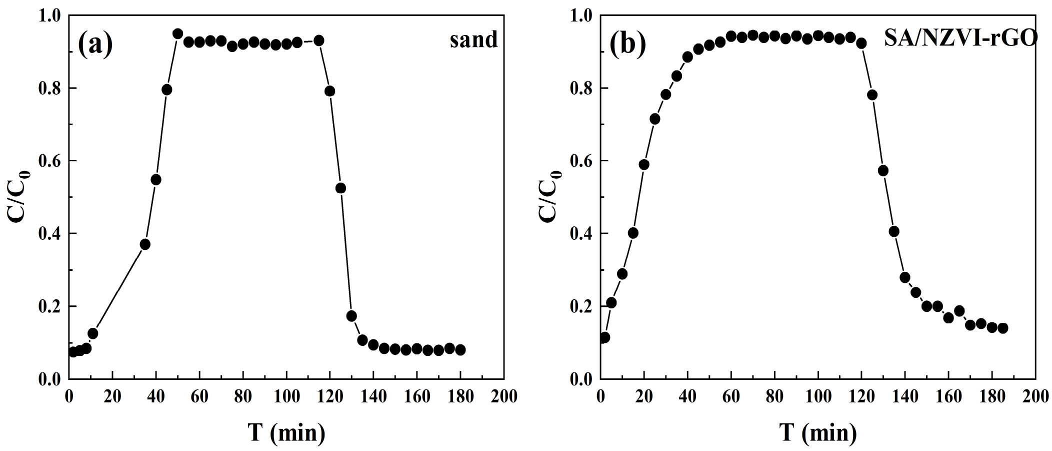

Figure S6. Breakthrough-leaching curves of KCl in quartz sand (a) and SA/NZVI-rGO (b).

Table S2. Parameters of the packed quartz sand columns.

Parameter	Value
Column length (L)	8.0 cm
Column diameter (d)	2.0 cm
Size of sands	16-30 mesh
Pore Volume (PV)	11.30 ml
Porosity	0.38
Permeability coefficient (K_s)	0.498 cm/s
Dispersion coefficient (D)	0.705 cm^2/min
Bulk density (ρ)	1.38 g/cm^3

Table S3. Parameters of the packed SA/NZVI-rGO gel beads columns.

Parameter	Value
Column length (L)	6.0 cm
Column diameter (d)	2.0 cm
Porosity	0.511
Permeability coefficient(K_s)	0.072 cm/s
Dispersion coefficient (D)	0.968 cm^2/min
Bulk density (ρ)	0.075 g/cm^3

Text S6. Mass Recovery Protocol

After the migration experiments, the porous medium was dissected into four sections from top to bottom, and each section was removed in turn into a 100 mL brown bottle and shaken vigorously for 30 min by adding 20 mL of background solution to release the microplastics trapped in the filled medium for sample concentration analysis. To obtain the mass balance for each migration experiment, the sample effluent mass was obtained by integrating the penetration-drench curve (effluent concentration as the vertical coordinate and sample inflow volume as the horizontal coordinate) and then dividing by the total sample inflow mass to obtain the percentage of sample flowing out of the column. The percentage of sample recovered from the porous media was obtained by dividing the mass of sample recovered from the porous media, by the mass of the total sample injected. The percentages of retained sample and sample passing through the column were added to give the overall recovery of the sample (mass balance) (Table S4).

Table S4. The mass balances of MPs and Cr(VI) in quartz sand column at experimental conditions.

System	pH	IS (mM)	Mass Balance		
			eff (%)	ret (%)	rec (%)
Only MPs	5	5	60.39	11.31	71.70
	7	5	68.85	5.61	74.43
	7	25	58.81	14.15	72.96
Co-transport MPs	5	5	49.99	23.24	73.23
	7	5	56.06	21.26	77.32
	7	25	43.77	22.33	66.10
Only Cr(VI)	5	5	81.88	2.20	84.08
	7	5	86.11	2.09	88.20
	7	25	85.67	2.15	87.82
Co-transport Cr(VI)	5	5	82.74	1.97	84.71
	7	5	87.52	2.02	89.54
	7	25	86.09	1.59	87.68

% eff refers to the percentage of MPs / Cr(VI) passed through columns.

% ret refers to the percentage of MPs / Cr(VI) retained in columns.

% rec refers to the total percentage of MPs / Cr(VI) recovered from columns.

Text S7. Batch experiments

The removal of Cr(VI) (20 mg/L) and MPs (5 mg/L) in single and double systems (100 mL) was compared by batch experiments with beads (0.5 g) under different experimental conditions. All adsorption experiments were carried out in a water bath thermostatic oscillator at 260 rad/min. Aliquots were removed at certain time intervals until the adsorption equilibrium. the removal rates of Cr(VI) and MPs were calculated as follows:

$$Removal(\%) = \frac{(C_0 - C_t)}{C_0} \times 100 \tag{S2}$$

where C_0 (mg/L) and C_t (mg/L)are the initial concentration and the concentration after a period t, respectively.

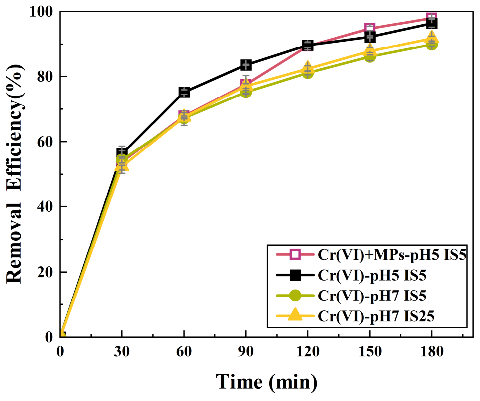

Figure S7. Removal efficiency of Cr(VI) in the absence and presence of MPs under different experimental conditions.

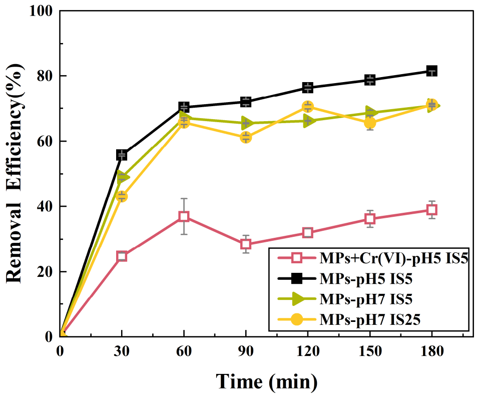

Figure S8. Removal efficiency of MPs in the absence and presence of Cr(VI) under different experimental conditions.

Text S8. Adsorption isotherm experiments

The adsorption isotherm elucidates the monolayer and multilayer intermolecular interactions of adsorbates on the adsorbent surface.The Langmuir isotherm model assumes that adsorption occurs on a uniform surface of the adsorbent, that the adsorption sites on the adsorbent have the same binding force to the contaminant, that it is a monolayer adsorption, and that the adsorption capacity is determined by the number of adsorption sites on the adsorbent.The linear and nonlinear equations of the Langmuir model take the form are as follows:

$$\frac{C_e}{Q_e} = \frac{1}{K_L Q_m} + \frac{C_e}{Q_m} \tag{S3}$$

$$Q_\mathrm{e} = \frac{Q_m K_L C_e}{1 + K_L C_e} \tag{S4}$$

where C_e (mg/L) and Q_e (mg/g) are the equilibrium concentration and equilibrium adsorption amount; K_L (L/mg) is a constant related to the adsorption energy; Q_m (mg/g) is the theoretical maximum adsorption amount.

The Freundlich isothermal model is an empirical model. It assumes that the adsorbent has a non-uniform adsorption surface with different binding forces at each adsorption site and is multilayered.The linear and nonlinear equations of the Freundlich model take the following form:

$$\ln Q_e = \ln k_F + \frac{\ln C_e}{n} \tag{S4}$$

$$Q_\mathrm{e} = K_F C_e^{\frac{1}{n}} \tag{S5}$$

where K_F is a constant related to the capacity of the adsorbent; n is a constant that responds to the adsorption strength of the adsorbent.

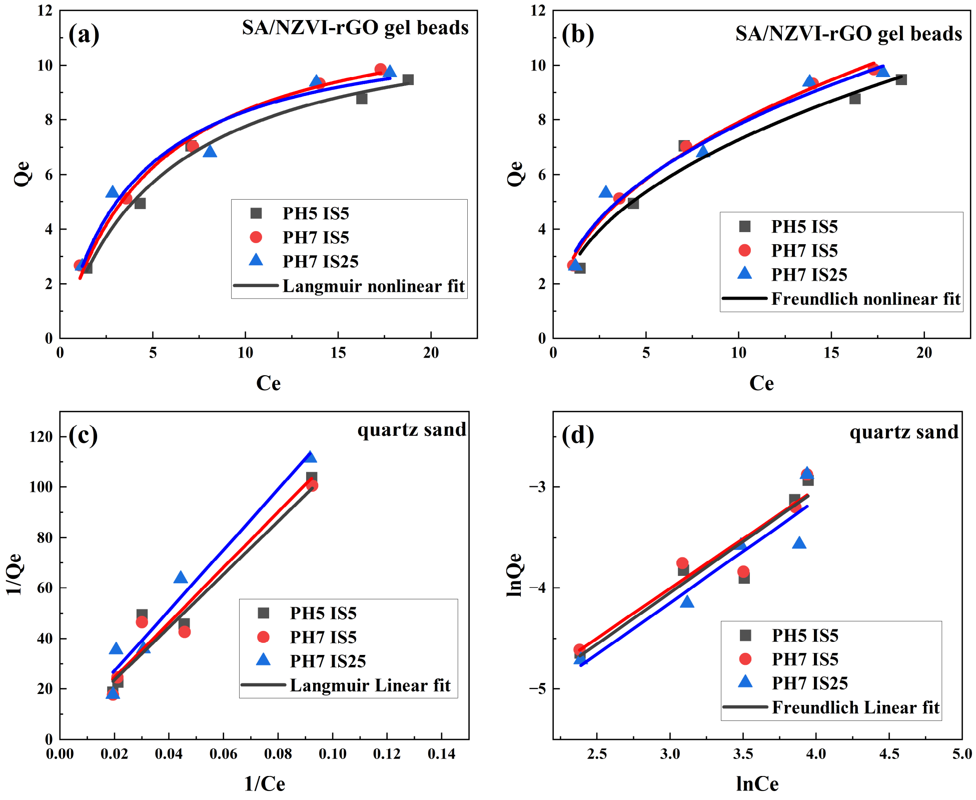

Figure S9. Langmuir and Freundlich isotherms for removing Cr(VI) under the coexistence system by SA/NZVI-rGO gel beads and quartz sand, respectively. Experimental conditions: concentration of Cr(VI) = 10–50 mg/L, concentration of MPs = 5 mg/L, and temperature = 16°C.

Text S9. The caculation of DLVO interaction

The classical Derjaguin-Landau-Verwey-Overbeek (DLVO) theory is a function of the dispersion distance and takes into account the polar Lifshitz-van der Waals (LW) attractive force and the electric double layer (EL) repulsive force, which is used to describe the interaction energy between spherical particles and surfaces [71]. The interaction between microplastic particles and quartz sand is predicted using this theory as a sphere-plate geometry system (equation S6) [72-73]. The following equations (S7) and (S8) were used to calculate the LW and EL between microplastic particles and quartz sand.

$$\Phi^{Total}(h) = \Phi_S^{LW}(h) + \Phi_S^{EL}(h) \quad \text{(S6)}$$

$$\Phi_S^{LW}(h) = -\frac{A_{123}a_P}{6h}\left(1+\frac{14h}{\lambda}\right)^{-1} \quad \text{(S7)}$$

$$\Phi_S^{EL}(h) = \pi a_P \varepsilon\varepsilon_0 \left\{2\zeta_p\zeta_s \, ln\left[\frac{1+exp(-\kappa h)}{1-exp(-\kappa h)}\right] + \left(\zeta_p^2+\zeta_s^2\right) ln[1-exp(-2\kappa h)]\right\}$$

(S8)

where h is the separation distance, a_P is the mean plastic particles radius. λ is the characteristic wavelength of the interaction (usually taken as 100 nm), The quantity A_{132} is the Hamaker constant for substances "1" and "2" in the presence of medium "3" and can be determined from the Hamaker constant of each material. The detailed calculation of A_{132} is given in the next section. ε_0 is the dielectric permittivity of the vacuum (8.854×10^{-12} C V^{-1} m^{-1}), ε is the dielectric constant of water (78.5), ζ_p and ζ_s is the zeta potential of plastic particles and sand respectively, κ is Debye-Huckel parameters.

$$\kappa = \left(\frac{2e^2N_AI}{\varepsilon_0\varepsilon_r k_BT}\right)^{1/2} \quad \text{(S9)}$$

where N_A Avogadro constant (6.022×10^{23}), I is ionic strength.

Text S10. Caculation of Hamaker Constants

The Hamaker constant for the interaction between plastic particle (1) and quartz sand (2) in water (3) is calculated by:

$$A_{123} = (\sqrt{A_{33}}-\sqrt{A_{22}})(\sqrt{A_{11}}-\sqrt{A_{22}}) \quad \text{(S10)}$$

where A_{11}, A_{22}, and A_{33} is the Hamaker constants of "1", "2", and "3" in vacuum, respectively required for use in these equations. The Hamaker constants for MPs (A_{11} = 6.60×10^{-20} J/m^2), for water (A_{33} = 3.70×10^{-20} J/m^2) and quartz sand (A_{22} = 8.86×10^{-20} J/m^2) were used to calculate the value of A_{132}.

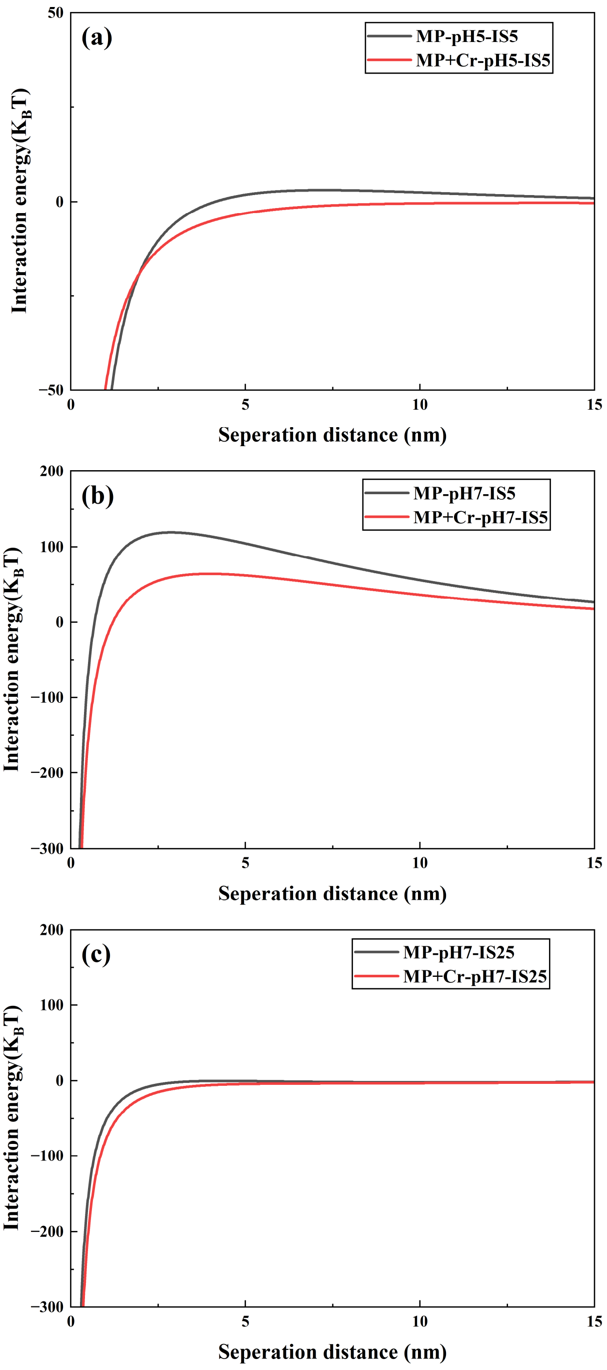

Figure S10. Interaction energy between MPs and quartz sand in the absence and presence of Cr(VI) at pH 5 IS 5 (a), pH 7 IS 5 (b) pH 7 IS 25 (c).

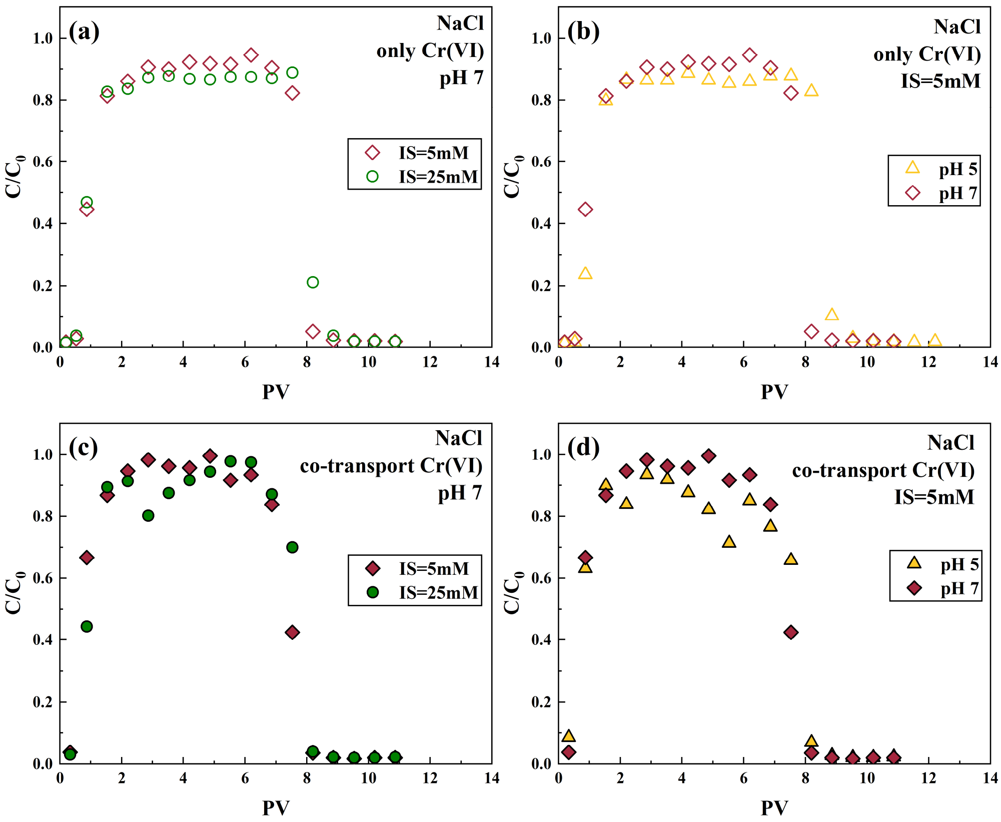

Figure S11. Breakthrough curves of Cr(VI) at different IS(a) and different pH(b) in the absence of MPs and at different IS(c) and different pH(d) in the presence of MPs.

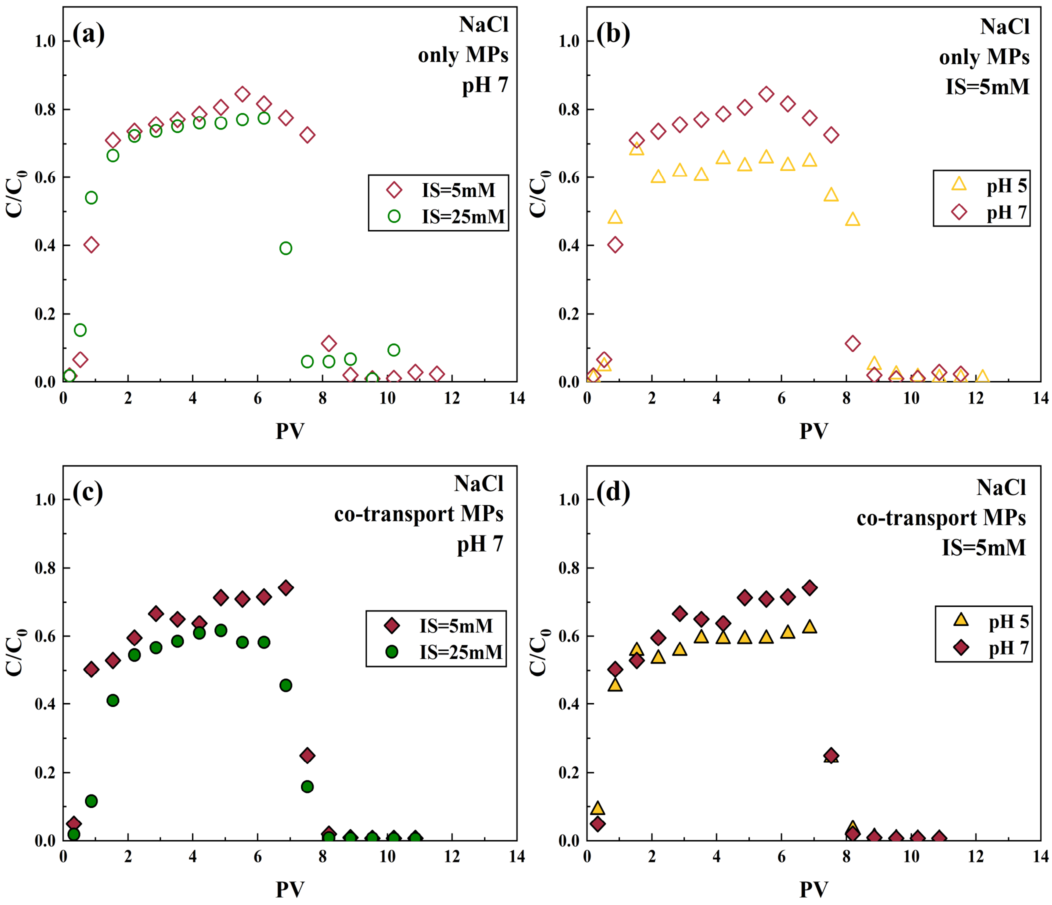

Figure S12. Breakthrough curves of MPs at different IS(a) and different pH(b) in the absence of Cr(VI) and at different IS(c) and different pH(d) in the presence of Cr(VI).

Reference

56. van Oss, C.J. Acid—Base Interfacial Interactions in Aqueous Media. *Colloids Surf. Physicochem. Eng. Asp.* **1993**, *78*, 1–49. https://doi.org/10.1016/0927-7757(93)80308-2.
70. Cai, L.; He, L.; Peng, S.; Li, M.; Tong, M. Influence of titanium dioxide nanoparticles on the transport and deposition of microplastics in quartz sand. *Environ. Pollut.* **2019**, *253*, 351–357. https://doi.org/10.1016/j.envpol.2019.07.006.
71. Bergendahl, J.; Grasso, D. Prediction of colloid detachment in a model porous media: Hydrodynamics. *Chem. Eng. Sci.* **2000**, *55*, 1523–1532. https://doi.org/10.1016/s0009-2509(99)00422-4.
72. van Oss, C.J. Hydrophobicity of biosurfaces—Origin, quantitative determination and interaction energies. *Colloids Surfaces B Biointerfaces Hydrophobicity* **1995**, *5*, 91–110. https://doi.org/10.1016/0927-7765(95)01217-7.
